# Association of Barrett's esophagus with obstructive sleep apnea syndrome: a bidirectional analysis of Mendelian randomization

**DOI:** 10.3389/fpsyt.2023.1269514

**Published:** 2024-01-05

**Authors:** Wei Tan, Yanli Cao, Liang Ge, Guangcai Li, Peijun Liu

**Affiliations:** ^1^Department of Respiratory and Critical Care Medicine, The Central Hospital of Enshi Tujia and Miao Autonomous Prefecture, Enshi, China; ^2^Hubei Selenium and Human Health Institute, The Central Hospital of Enshi Tujia and Miao Autonomous Prefecture, Enshi, China

**Keywords:** OSAS, Barrett's esophagus, Mendelian randomization, instrumental variables, GWAS

## Abstract

**Background:**

Observational studies have reported associations between Barrett's esophagus (BE) and obstructive sleep apnea syndrome (OSAS), but the causal relationship remained unclear due to potential confounding biases. Our study aimed to elucidate this causal relationship by deploying a two-sample Mendelian randomization (MR) methodology.

**Methods:**

Instrumental variables (IVs) for Barrett's esophagus were obtained from a public database that comprised 13,358 cases and 43,071 controls. To investigate OSAS, we utilized summary statistics from a comprehensive genome-wide association study (GWAS) encompassing 38,998 cases of OSAS and 336,659 controls. Our MR analyses adopted multiple techniques, including inverse variance weighted (IVW), weighted median, weighted mode, MR-Egger, and simple mode.

**Results:**

The IVW analysis established a causal relationship between Barrett's esophagus and OSAS, with an odds ratio (OR) of 1.19 and a 95% confidence interval (CI) of 1.11–1.28 (*p* = 8.88E-07). Furthermore, OSAS was identified as a contributing factor to the onset of Barrett's esophagus, with an OR of 1.44 and a 95% CI of 1.33–1.57 (*p* = 7.74E-19). Notably, the MR–Egger intercept test found no evidence of directional pleiotropy (*p* > 0.05).

**Conclusion:**

This study identifies a potential association between BE and an increased occurrence of OSAS, as well as the reverse relationship. These insights could influence future screening protocols and prevention strategies for both conditions.

## 1 Introduction

Barrett's esophagus (BE) is characterized by replacing normal squamous epithelial cells in the lower part of the esophagus with columnar epithelial cells (1). BE with intestinal metaplasia represents a significant concern as it is closely linked with a substantially elevated risk of progressing to esophageal cancer (2). Approximately 1–2% of the worldwide population is believed to have BE, primarily due to chronic gastroesophageal reflux (3, 4). While gastroesophageal reflux disease (GERD) is often associated with BE, it is vital to understand that BE can occur in its absence (5). The clinical manifestations of BE frequently include persistent heartburn, consistent acid reflux, dysphagia, and thoracic discomfort. Interestingly, some individuals with BE may not exhibit any symptoms (6). Obstructive sleep apnea syndrome (OSAS) is a prevalent sleep-related respiratory disorder impacting nearly a billion individuals globally. Its repercussions profoundly affect individual wellbeing and broader societal challenges (7). The hallmark of OSAS is the transient cessation or significant reduction of airflow during sleep. This phenomenon is primarily attributed to the relaxation of pharyngeal muscles, which leads to episodic airway obstructions (8). Clinically, OSAS is often accompanied by symptoms such as pronounced snoring, daytime lethargy, headaches upon awakening, diminished cognitive focus, and memory impairment (9). Interestingly, recent studies have revealed a bidirectional relationship between OSAS and BE. Lindam et al. demonstrated that individuals with excessive daytime sleepiness or symptoms related to sleep apnea show a higher prevalence of BE (10). Hadi et al. indicate that the risk of developing BE increases with the severity of OSAS, categorized in increments of 10 on the apnea-hypopnea index scale (11). Although there is an association between OSAS and BE, this association may either be mediated by GERD or independent of gastroesophageal reflux (12). Given the complexity of confounding factors such as GERD, any potential link between OSA and BE appears to have not been sufficiently explored.

GERD is commonly believed to be associated with OSAS and BE, but some studies and theories consider other potential pathways of connection (13). A prevailing hypothesis posits that OSAS might influence BE progression by initiating inflammatory cascades (14). It is well-acknowledged that OSAS is characterized by intermittent hypoxia, occurring due to periodic episodes of hypoxia followed by reoxygenation during sleep, which is associated with systemic inflammation (15). Elevated systemic inflammatory markers, observed in OSAS patients, are theorized to facilitate the onset of BE by inducing cellular injury, genetic aberrations, and heightened oncogenic risk (16). The main symptoms of BE include reflux symptoms such as heartburn and retrosternal pain, which may worsen at night, affecting sleep quality and potentially causing or exacerbating symptoms of OSAS (17, 18). Nevertheless, the current body of evidence does not conclusively indicate that BE directly aggravates or engenders OSAS.

Mendelian randomization (MR) stands as a robust analytical approach in observational research, seeking to elucidate causal associations between modifiable risk factors and disease outcomes by leveraging established functional genetic variants (19). There are numerous approaches within the two-sample methodology, and recently, the Mixture model Reciprocal Causation Inference (MRCI) has emerged as a novel statistical framework (20). Two-sample Mendelian randomization has emerged as a pivotal method within genetic epidemiology. This refers to a type of instrumental variable analysis in which genetic variations, notably single nucleotide polymorphisms (SNPs), serve as the instrumental variables. By employing known genetic markers, especially SNPs, as instrumental variables, this approach facilitates the estimation of causal associations between an exposure and a given outcome (21). Our study was designed to clarify the causal relationship between OSAS and BE by employing a two-sample MR approach.

## 2 Methods

### 2.1 Study design

**Figure 1:**
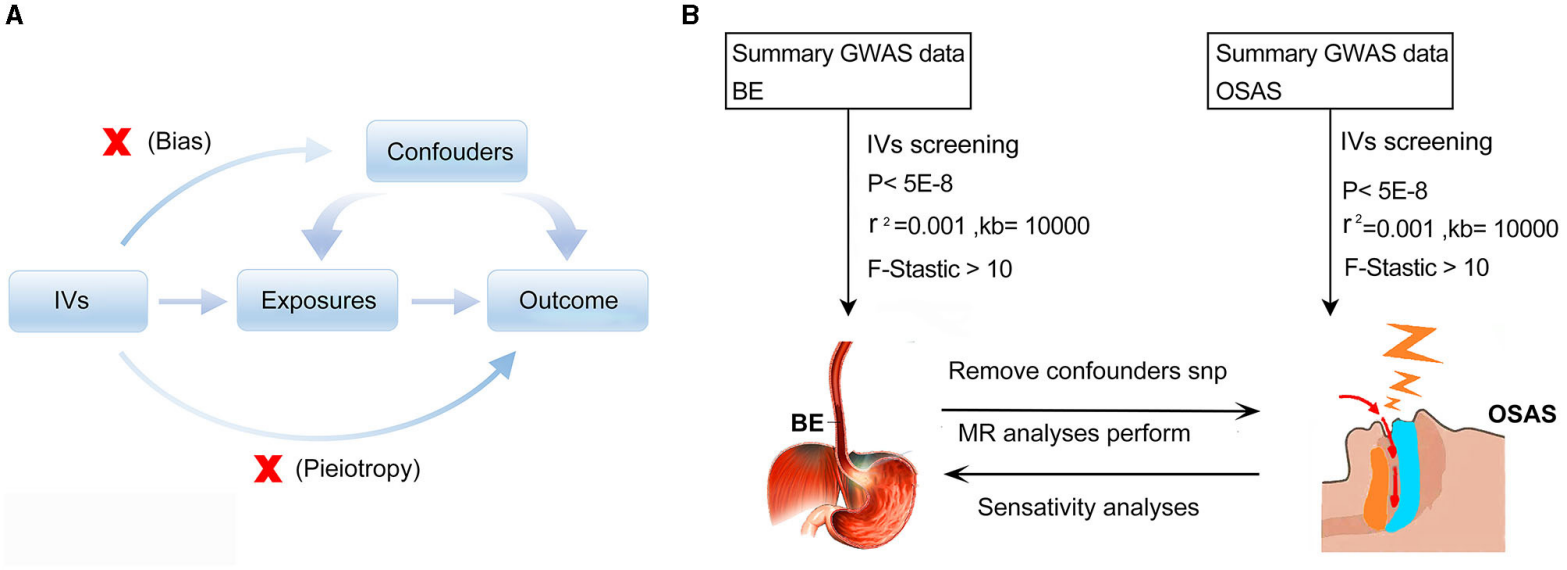
Schematic representations of the employed methodologies. **(A)** Illustration of Mendelian randomization principles. **(B)** Schematic diagram depicting the bidirectional Mendelian randomization methodology utilized to investigate the causal relationship between BE and OSAS. MR, Mendelian randomization; GWAS, genome-wide association study; IVs, Instrumental variables; BE, Barrett's esophagus; OSAS, Obstructive Sleep Apnea Syndrome.

The MR is underpinned by three cardinal assumptions: (1) the Instrumental variables (IVs) manifest a potent association with the exposure; (2) These IVs remain uncontaminated by potential confounders; and (3) the influence of the IVs on the outcome is channeled exclusively through their affiliation with the exposure, eschewing any ancillary pathways (22) (Figure 1A). The bidirectional Mendelian randomization methodology was executed to decipher the causative nexus between BE and OSAS, as delineated in Figure 1B.

### 2.2 Sources of data and selection of SNPs as IVs

Summary statistics of BE phenotypes were procured from the IEU GWAS database (https://gwas.mrcieu.ac.uk/datasets/ebi-a-GCST90000515/). The diagnosis of BE is established based on the ICD-10 code K22.7, primarily determined through a combination of patient self-reports and clinical diagnostic evaluations. This repository houses data from a cohort of 56,429 individuals of European descent, encompassing 13,358 diagnosed BE cases juxtaposed against 43,071 control subjects (23). For OSAS, pertinent genetic instruments were derived from the FinnGen database (https://storage.googleapis.com/finngen-public-data-r9/).

The diagnosis of OSAS is indicated by ICD-10 code G47.3. The confirmation of OSA involves the assessment of clinical symptoms and signs, along with the Apnea-Hypopnea Index (AHI) of ≥5 events per hour. This collection incorporates an aggregation of 38,998 OSAS cases and 336,659 control subjects. It's paramount to emphasize that the FinnGen dataset originates from individuals of European lineage (24).

In the process of choosing suitable instrumental variables, SNPs are required to fulfill a range of criteria. The selection of IVs necessitated SNPs to meet a rigorous set of criteria. Initially, SNPs must manifest a prominent association with the exposure on a genome-wide scale. This dictates that a statistically significant relationship between the SNPs and the exposure should be established, adhering to a *p*-value threshold of <5E-08. This stringent criterion bolsters confidence in the integrity of SNP exposure associations, mitigating potential false-positive results (25). Second, SNPs selection hinged on the PLINK algorithm to identify SNPs not in linkage disequilibrium, typified by an *r*^2^ < 0.001. Within a clumping window of 10,000 kilobases, this procedure is designed to precisely identify genuinely independent SNPs, eliminating potential correlations from nearby genetic markers. Third, A meticulous curation was executed utilizing the PhenoScanner GWAS database (http://phenoscanner.medschl.cam.ac.uk) to omit IVs conceivably linked to the investigative outcome trait, including those potentially associated with GERD, smoking, and alcohol consumption (26). SNPs registering an F-Statistic < 10 were earmarked as weak IVs and were consequently sidelined from subsequent analyses (27). Ultimately, 8 SNPs were identified as instrumental variables for OSAS, and 11 SNPs emerged as instrumental variables for BE. This deliberate exclusion strategy was orchestrated to ensure the incorporation of robust and dependable IVs in the MR paradigm. In conclusion, the selection process systematically excluded palindromic SNPs and those demonstrating associations with the outcome at a genome-wide significance threshold.

### 2.3 Genetic correlation analysis

To assess the genetic correlations between BE and OSAS, our study employed the Linkage Disequilibrium Score Regression (LDSC) software package, accessible at (https://github.com/bulik/ldsc) (28). This methodological approach was instrumental in quantifying the extent of genetic overlap and potential shared etiological pathways between these two conditions.

### 2.4 Evaluation of OSAS and BE data overlap

To strictly adhere to established guidelines, we meticulously assessed the sample overlapping ratio between OSAS and BE (29). Our analysis showed that the BE GWAS data, derived from prominent databases including the Barrett's and Esophageal Adenocarcinoma Consortium (BEACON), as well as the comprehensive datasets from Bonn, Cambridge, Oxford, and UKB, did not display potential overlap with the FinnGen database for OSAS (23, 30, 31). The observation reinforces the integrity of our two-sample MR approach.

### 2.5 Analyses based on Mendelian randomization

This investigation harnessed an array of MR methodologies, notably IVW, MR-Egger, weighted median, weighted mode, and simple mode, intending to elucidate the causative linkage between BE and OSAS (29). To leverage its higher statistical power compared to other MR methods, the IVW approach, assuming the validity of all SNPs used as IVs, was selected as this research's primary analysis and main analytical approach (32). This method capitalizes on the vigor of the genetic correlation IVs, amalgamating the precision of each estimation. Consequently, enhanced emphasis is accorded to more precise measures, facilitating a causal extrapolation between the exposure and the resultant outcome. Subsidiary methods acted in tandem with IVW, each entrenched in its unique set of assumptions concerning horizontal pleiotropy, with a collective objective to provide comprehensive and resilient MR estimates across various contexts.

### 2.6 Statistical analysis and sensitivity assessments

We performed statistical analyses with R software (version 4.1.2) and the “Two Sample MR” package. Cochran's *Q*-test, integral to the IVW methodology, probes the heterogeneity across instrumental variables. The *p*-value falling below 0.05 typically flags significant disparities across the scrutinized cohorts. Pleiotropy embodies the intriguing paradigm wherein an isolated gene influences multiple traits. In deciphering horizontal pleiotropy and pinpointing anomalous variants, the study turns to the MR Pleiotropy RESidual Sum and Outlier PRESSO methodology (33). Discarding these divergent SNPs generates a decontaminated causal inference via the outlier-corrected Mendelian randomization assessment. The research employs the MR-Egger intercept examination to probe directional pleiotropy within the IVs. An intercept test bearing a non-zero magnitude intimates the manifestation of directional pleiotropy in the set of IVs (34). A leave-one-out diagnostic is orchestrated for a granular assessment of potential SNP-driven biases in MR outcomes, wherein each SNP is iteratively sidelined, and the consequent ramifications on analytical outputs are scrutinized (35).

## 3 Results

### 3.1 Genetic correlation analysis of BE and OSAS

In our analysis utilizing the LDSC methodology, we observed a notable genetic correlation between BE and OSAS. The results indicated a significant correlation coefficient (rg) of 0.35, with a highly significant *p*-value of 1.36e-20 (Table 1).

**Table 1:** Genetic correlation estimates from LDSC regression.

**Phenotype 1**	**Phenotype 2**	**Rg (SE)**	**Oval**
BE	OSAS	0.35 (0.03)	1.36e-20

BE, Barrett's esophagus; OSAS, obstructive sleep apnea syndrome; Rg, genetic correlation; Pval, the p-value for rg; SE, the standard error of Rg.

### 3.2 The influence of OSAS on the phenomenon of BE

**Figure 2:**
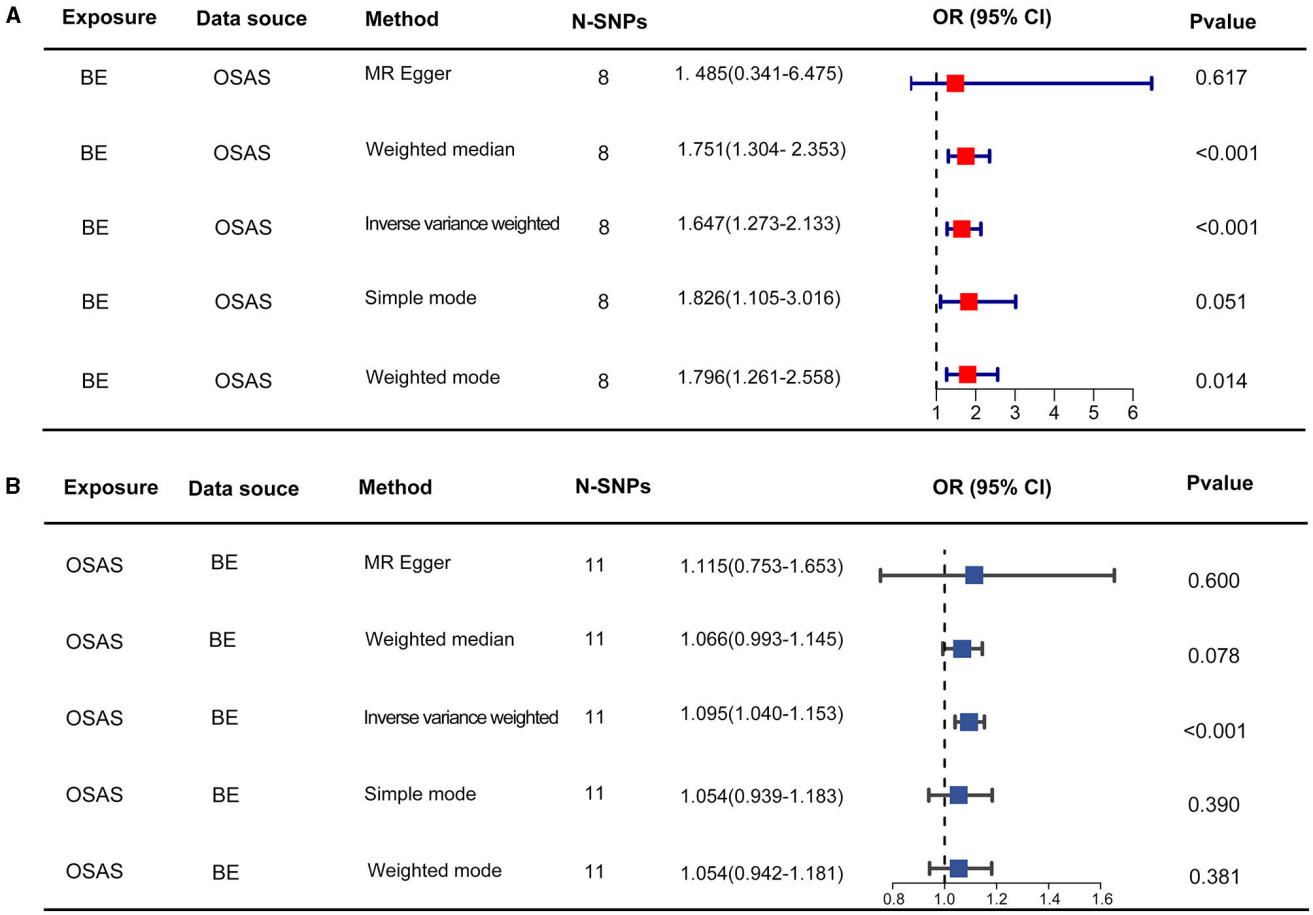
The results of two-sample MR assessing causal relationships between OSAS and BE. **(A)** Represents the causal estimates of OSAS influencing BE. **(B)** Illustrates the causal estimates of BE affecting OSAS. MR, Mendelian randomization; OSAS, obstructive sleep apnea syndrome; BE, Barrett's esophagus; OR, odds ratio.

**Figure 3:**
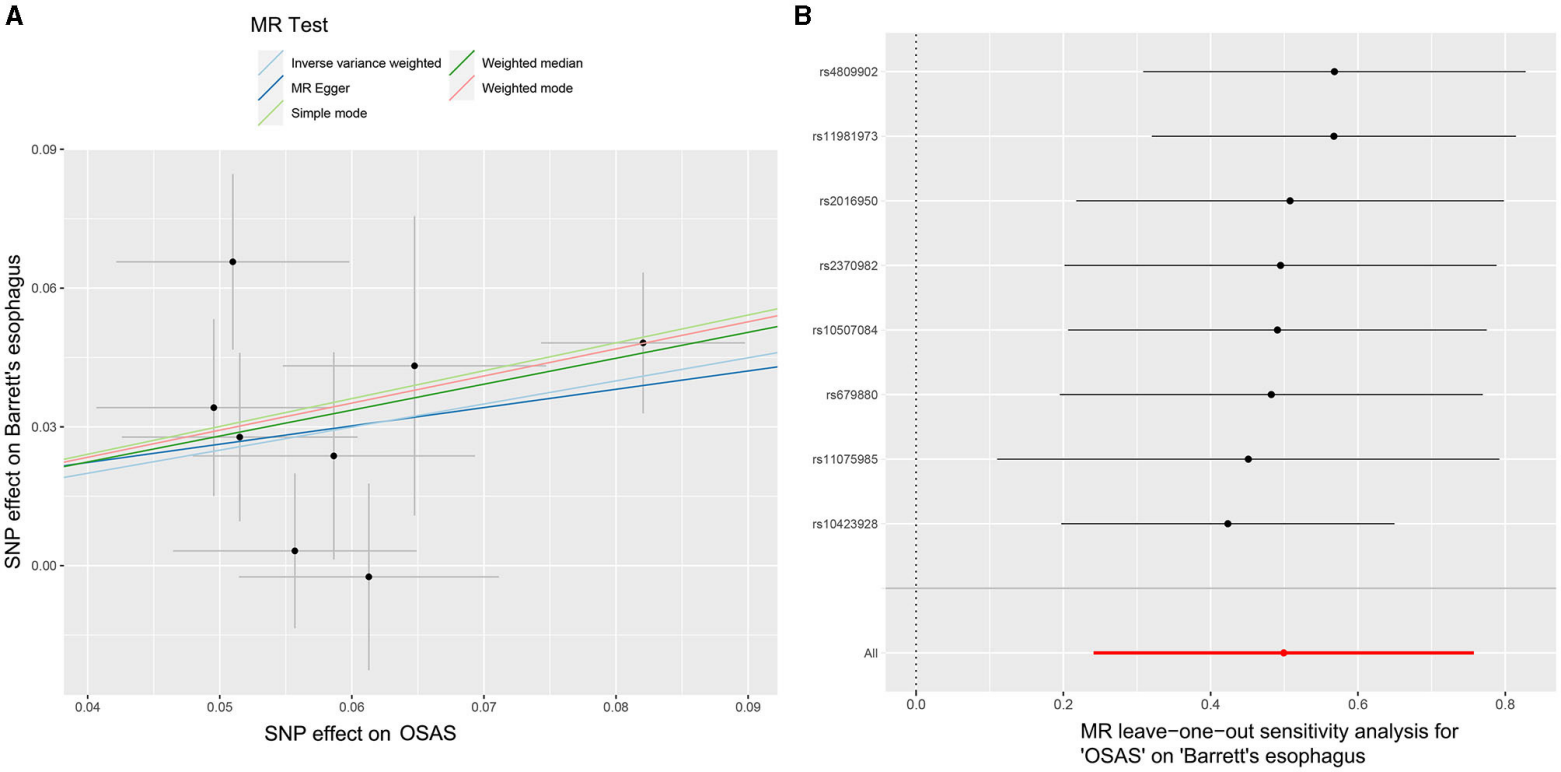
MR analyses illustrating the causal relationship between OSAS as the exposure and BE as the outcome. **(A)** Scatter plot illustrating individual SNP analyses investigating the impact of OSAS on BE incidence. **(B)** Leave-one-out plot showing the collective influence of SNPs on the relationship between OSAS and BE. MR, Mendelian randomization; OSAS, obstructive sleep apnea syndrome; BE, Barrett's esophagus; SNP, single nucleotide polymorphism.

Initially, SNPs manifesting a notable association with OSAS at the genome-wide threshold were delineated, with a concurrent emphasis on SNPs devoid of linkage disequilibrium. After meticulously excluding SNPs that exhibited pleiotropic tendencies, such as those related to reverse causation, reflux, and obesity, among other factors, a refined set of 8 SNPs emerged as IVs. These SNPs were characterized by an F-Statistic exceeding 10, underscoring their robust instrument strength (Supplementary Table 1). Employing the IVW method yielded an odds ratio (OR) of 1.647 (95% CI: 1.273–2.133, *p* = 0.00015). Similarly, the weighted median method produced an OR of 1.751 (95% CI: 1.304–2.353, *p* = 0.00015), while the weighted mode method indicated an OR of 1.796 (95% CI: 1.261–2.558, *p* = 0.021). These findings robustly intimate a causal linkage between Barrett's esophagus (BE) and the onset of OSAS. In stark contrast, outcomes from the MR-Egger method (OR: 1.485, 95% CI: 0.341–6.475, *p* = 0.617) and the simple mode (OR: 1.83, 95% CI: 1.105–3.016, *p* = 0.051) refrained from corroborating a causal relationship between BE and OSAS (Figure 2A). The IVW method presents a distinct merit, adeptly managing multiple SNPs concurrently and maintaining its efficacy even amidst tenuous correlations between SNPs (36). Significant heterogeneity was evident, as indicated by Cochran's *Q*-test (*Q* = 9.989, *p* = 0.0292). The MR-Egger intercept analysis underscored a lack of directional pleiotropy (*p* = 0.893; Table 2). Scatterplots detailing the outcomes of these evaluations are shown in Figure 3A. The leave-one-out assessment revealed that no singular SNP predominantly swayed the overarching influence of BE (as the exposure) on the incidence of OSAS (as the outcome), as depicted in Figure 3B.

**Table 2:** OSAS and BE: pleiotropy and heterogeneity analysis.

**Exposure**	**Outcome**	**Heterogeneity**		**MR-egger intercept**	
		**Cochrane's** ***Q***	**Heterogeneity (pval)**	**Egger-intercept**	**Pleiotropy (pval)**
OSAS	BE	9.989	0.0292	0.0064	0.893
BE	OSAS	8.893	0.0442	−0.0017	0.929

OSAS, obstructive sleep apnea syndrome; BE, Barrett's esophagus; MR, Mendelian randomization.

### 3.3 The impact of BE on the occurrence of OSAS

**Figure 4:**
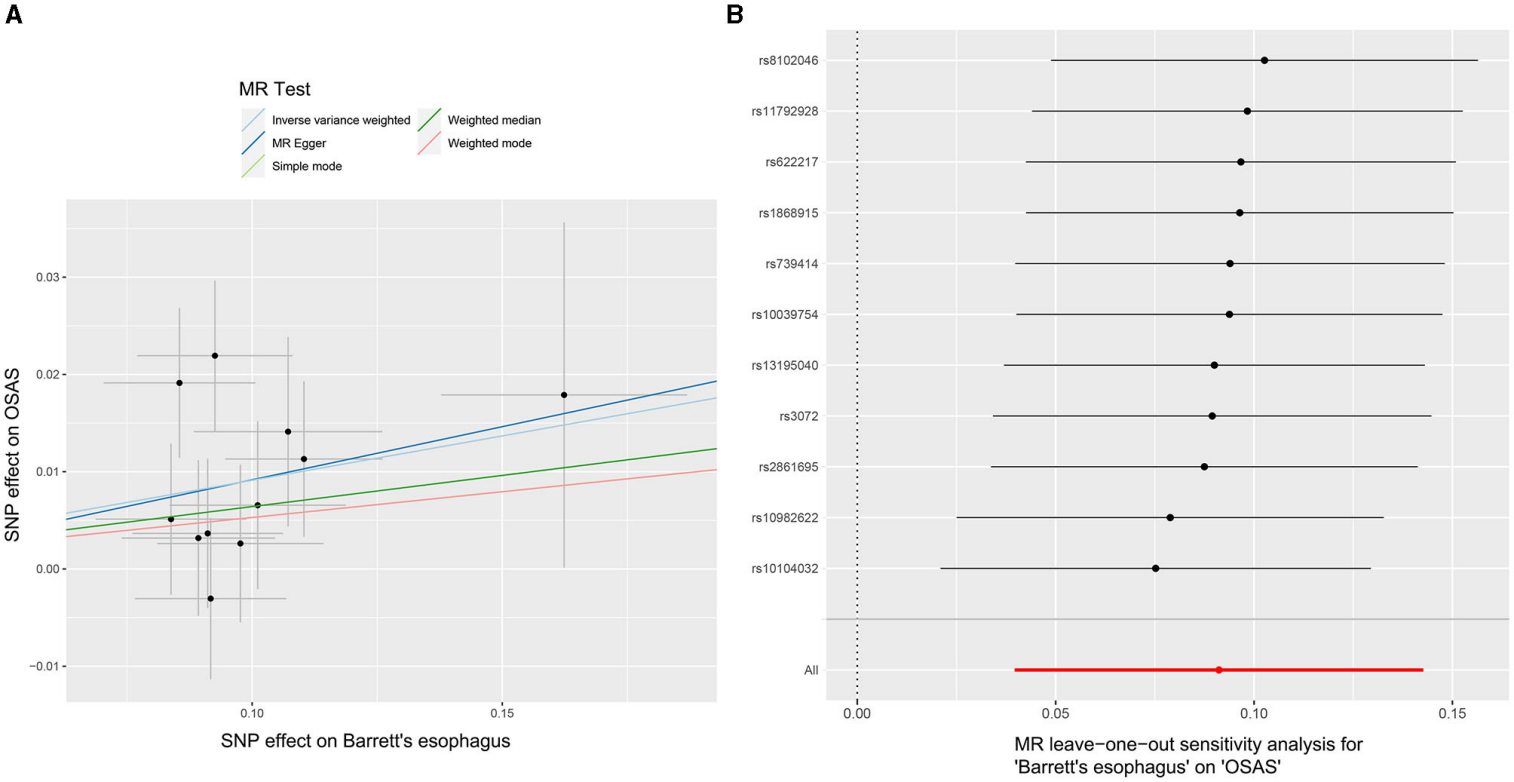
MR analyses demonstrating the causal relationship with BE as the exposure and OSAS as the outcome. **(A)** Scatter plot illustrating individual SNP analyses investigating the influence of BE on the prevalence of OSAS. **(B)** Leave-one-out plot demonstrating the combined impact of SNPs on the relationship between BE and OSAS. MR, Mendelian randomization; BE, Barrett's esophagus; OSAS, obstructive sleep apnea syndrome; SNP, Single Nucleotide Polymorphism.

Through our rigorous analysis, we discerned specific SNPs that exhibited genome-wide notable associations with BE, ensuring the inclusion of those not found in linkage disequilibrium. After meticulous screening to exclude pleiotropic SNPs linked with obesity, smoking, and other potential confounders, a concise set of 11 SNPs emerged as IVs, as detailed in Supplementary Table 2. The results derived from the IVW method offered compelling evidence, pointing to a reliable causal link between OSAS and BE, as underscored by an OR of 1.095 (95% CI: 1.040–1.153, *p* = 0.00052). In contrast, other analytical methods yielded disparate results. While the weighted median process suggested an OR of 1.066 (95% CI: 0.993–1.145, *p* = 0.078), the weighted mode method produced an OR of 1.054 (95% CI: 0.942–1.181, *p* = 0.381). Moreover, the MR-Egger approach showed an OR of 1.115 (95% CI: 0.753–1.653, *p* = 0.6), and the simple mode method indicated an OR of 1.054 (95% CI: 0.939–1.183, *p* = 0.390). These findings, encapsulated in Figure 2B, did not conclusively affirm a causal association between OSAS and BE. Significant heterogeneity across the involved studies emerged, as illuminated by Cochran's *Q*-test results (*Q* = 8.893, *p* = 0.0442), pinpointing variances in effect estimates across disparate datasets. However, the MR-Egger intercept analysis suggested a lack of directional pleiotropy (*p* = 0.929), implying minimal to no influence of horizontal pleiotropic effects on the MR results. These fundamental observations are illustrated in Figure 4A. The subsequent leave-one-out assessment consistently revealed that none of the individual SNPs significantly impacted the overall association between OSAS exposure and the incidence of BE, as depicted in Figure 4B.

## 4 Discussion

This is the first comprehensive study delving into the nuanced relationship between OSAS and BE through the two-sample Mendelian randomization approach. Our study, conducted among individuals of European ancestry, provides compelling evidence of a causal relationship between OSAS and BE, highlighting the interconnection between these seemingly unrelated conditions. Amidst rising healthcare challenges, our findings shed fresh light on the underlying mechanisms linking OSAS and BE, offering pivotal insights that could pave the way for enhanced diagnostic precision and therapeutic interventions.

The objective of our study was to investigate the causal association between OSAS and BE using multiple MR methods. We employed the IVW method as the primary approach, supplemented by weighted median and mode methods. Our findings indicated a causal link from OSAS to BE according to these methods. Interestingly, the IVW analysis also suggested that BE may causally influence the development of OSAS. However, it is important to note that the MR-Egger method did not provide support for a causal relationship in either direction. This discrepancy highlights the complexity inherent in MR research. The IVW method, commonly used in two-sample MR studies, generates an overall estimate of the causal effect when instrumental variables are robust and unaffected by pleiotropy (37). MR-Egger is specifically designed to address biases arising from pleiotropy but can be overly cautious, potentially leading to an underestimation of true effects. To account for potential pleiotropy, we incorporated the MR-Egger intercept in our analysis, following established methodologies. Based on our analysis, and following the methodology adopted in various literature sources, we considered the IVW as the primary approach for our research (38–40).

Earlier investigations have highlighted a pronounced augmentation in the likelihood of OSAS onset in individuals diagnosed with BE (41). Despite prior indications, the effects of OSAS on BE have been sparingly explored. Delving deeper into this association's genomic intricacies and clinical implications is paramount, especially when considering potential confounders such as obesity, smoking, and reflux. In this context, our study harnessed the power of a two-sample Mendelian randomization approach to probe the nexus between OSAS and BE. By deploying MR analysis, we adeptly sidestepped the pitfalls of confounding elements and the perils of reverse causation that often plague observational studies. Furthermore, this method alleviates the burdens of exorbitant expenses and logistical challenges typically affiliated with randomized controlled trials. The prevailing guidelines advocate for screening individuals deemed high-risk for BE, an antecedent to esophageal cancer (42). While both BE and OSAS exhibit similarities in common risk factors, such as elevated body mass index (BMI) and GERD, the exact correlation between these conditions still requires further investigation and conclusive determination (17, 43).

In comparison to individuals without OSAS, patients afflicted with OSAS exhibited a heightened risk of developing Barrett's esophagus (*p* < 0.001, OR:3.26,95% CI: 1.72–6.85) (44). With the severity of OSAS, there is a heightened risk of BE, setting the AHI of 10 as the critical marker. In a distinct multivariable regression analysis, where OSAS was delineated based on 10-point increments in the AHI, a notable increase in BE risk was discerned with every 10-point rise in AHI (OR 1.10, 95% CI: 1.02–1.19). While GERD plays a pivotal role in the onset of BE among OSAS sufferers, the observed correlations could also be amplified by other underlying factors, such as pronounced central obesity (marked rise in visceral abdominal fat) and a prevailing systemic inflammatory condition (45). Hence, an intricate interplay of factors could underpin the heightened prevalence of BE in OSAS patients.

A meta-analysis of six studies, including 2,333 patients who met the inclusion criteria, demonstrated a significant elevation in the risk of OSAS, a high risk of OSAS, and the presence of patient-reported OSAS symptoms in individuals with Barrett's esophagus compared to those without this condition. The combined OR was 2.19 (95% CI: 1.53–3.15), indicating a significant association between BE and OSAS. Additionally, a subgroup analysis comprising two studies focusing on cases with confirmed OSA through polysomnography and Barrett's esophagus demonstrated a significant association, with an OR of 2.59 (95% CI: 1.39–4.84) (41). In previous notions, it was postulated that BE escalates the prevalence of OSAS, potentially due to its association with obesity. However, a recent study has reported that the correlation between BE and OSAS remains unaltered, even in the presence of obesity-related factors (46).

In this MR investigation, we harnessed genetic datasets spanning various European nations to delve into the interrelation between OSAS and BE. By leveraging large case-control samples and rigorous statistical analysis, our findings confirm a significant association between these two conditions and unveil a bidirectional causal relationship. This outcome reinforces that OSAS could be instrumental in BE onset and vice versa, underscoring a multifaceted dynamic between these medical conditions. The intricacies of the BE and OSAS connection remain elusive, yet prevailing theories posit the involvement of mechanical stressors, neural reflex arcs, and inflammation-induced pathways (11). BE may impose pressure on adjacent tissues, potentially inducing airway blockage and heightening OSAS susceptibility. Furthermore, it might interfere with respiratory regulation through neural feedback loops bridging the esophageal and respiratory channels. Inflammatory cascades activated by BE-induced damage could unleash agents detrimental to respiratory and muscular structures, thereby facilitating OSAS. The Cochran Q test (*p* < 0.05) suggests the presence of heterogeneity. However, it does not invalidate the validity of the IVW estimates under the random-effects model, even in the presence of heterogeneity. Additionally, the MR-Egger intercept analysis indicated no directional pleiotropy, indicating minimal potential for measurement error or bias in the selected instrumental variables. These factors collectively support the robustness and credibility of the MR findings.

Nonetheless, it should be noted that our study does have certain limitations. Not all Mendelian Randomization methods support a causal relationship between OSA and BE. We may consider conducting clinical randomized controlled trials (RCTs) in future studies to further investigate this relationship. Due to the reliance on publicly accessible summary statistics, we could not access detailed demographic data, such as age and gender, which represents a limitation in our study. However, the essence of Mendelian randomization is to leverage genetic instrumental variables to discern potential causal relationships between exposures and outcomes, even without specific demographic details. Despite these limitations, our study contributes to the field by using genetic information to explore causal associations.

## 5 Conclusion

The relationship between OSA and BE is complex and not fully understood, as the current MR analysis indicates. However, research suggests a potential role of OSA in BE pathogenesis and vice versa, implying a bidirectional influence. This has implications for mitigating the risk of both conditions. When managing OSA, consider gastrointestinal symptoms and conduct pH monitoring or endoscopy if BE is suspected. Non-pharmacological interventions such as diet control, weight loss, and reducing alcohol consumption may be effective for BE patients. Screening for undiagnosed OSA in BE patients is essential.

## Data availability statement

The original contributions presented in the study are included in the article/Supplementary material, further inquiries can be directed to the corresponding authors.

## Author contributions

WT: Investigation, Methodology, Writing – original draft. YC: Resources, Software, Writing – original draft. LG: Data curation, Resources, Software, Writing – review & editing. GL: Investigation, Supervision, Writing – review & editing. PL: Conceptualization, Methodology, Project administration, Supervision, Validation, Writing – review & editing.
