# Supplementary material for: Association of Barrett's esophagus with obstructive sleep apnea syndrome: a bidirectional analysis of Mendelian randomization
Source: Front Psychiatry. 2024 Jan 5;14:1269514. doi: 10.3389/fpsyt.2023.1269514 (PMC10796615; doi:10.3389/fpsyt.2023.1269514)
Supplement: Supplementary file 1 [file Table_1.docx]

| SNP | effect_allele | other_allele | beta.exposure | beta.outcome | eaf.exposure | eaf.outcome | se.exposure | chr | samplesize | outcome | exposure | pval |
| --- | --- | --- | --- | --- | --- | --- | --- | --- | --- | --- | --- | --- |
| rs10423928 | A | T | -0.050987 | -0.0656811 | 0.258578 | 0.19173 | 0.00882387 | 19 | 56429 | Barrett's esophagus | OSAS | 7.55E-09 |
| rs10507084 | T | C | 0.0647465 | 0.0431972 | 0.179407 | 0.054396 | 0.00996658 | 12 | 56429 | Barrett's esophagus | OSAS | 8.23E-11 |
| rs11075985 | A | C | 0.0820534 | 0.0481568 | 0.428739 | 0.424439 | 0.00771867 | 16 | 56429 | Barrett's esophagus | OSAS | 2.15E-26 |
| rs11981973 | G | A | 0.061286 | -0.00242124 | 0.181587 | 0.164638 | 0.00984022 | 7 | 56429 | Barrett's esophagus | OSAS | 4.72E-10 |
| rs2016950 | T | C | -0.0586327 | -0.0237212 | 0.157983 | 0.132414 | 0.0106896 | 12 | 56429 | Barrett's esophagus | OSAS | 4.13E-08 |
| rs2370982 | T | C | 0.0515131 | 0.0277902 | 0.238198 | 0.214336 | 0.00892902 | 14 | 56429 | Barrett's esophagus | OSAS | 7.97E-09 |
| rs4809902 | C | G | -0.0556845 | -0.00319034 | 0.228498 | 0.28019 | 0.00921496 | 20 | 56429 | Barrett's esophagus | OSAS | 1.51E-09 |
| rs679880 | A | G | 0.0495476 | 0.0341575 | 0.745023 | 0.803905 | 0.00889332 | 9 | 56429 | Barrett's esophagus | OSAS | 2.53E-08 |

**TableS1** **|** **Comprehensive details of the SNPs utilized in the MR analysis of OSAS on BE**
