# Supplementary material for: Association of Barrett's esophagus with obstructive sleep apnea syndrome: a bidirectional analysis of Mendelian randomization
Source: Front Psychiatry. 2024 Jan 5;14:1269514. doi: 10.3389/fpsyt.2023.1269514 (PMC10796615; doi:10.3389/fpsyt.2023.1269514)
Supplement: Supplementary file 2 [file Table_2.docx]

| SNP | effect_allele | other_allele | beta.exposure | beta.outcome | eaf.exposure | eaf.outcome | se.exposure | chr | samplesize | outcome | exposure | pval |
| --- | --- | --- | --- | --- | --- | --- | --- | --- | --- | --- | --- | --- |
| rs10039754 | G | A | 0.0837988 | 0.00511915 | 0.550136 | 0.570561 | 0.0150523 | 5 | 56429 | OSAS | Barrett's esophagus | 2.59E-08 |
| rs10104032 | A | C | 0.0925762 | 0.021925 | 0.374883 | 0.479518 | 0.0155502 | 8 | 56429 | OSAS | Barrett's esophagus | 2.63E-09 |
| rs10982622 | G | A | -0.0854904 | -0.0191276 | 0.461742 | 0.544708 | 0.0151732 | 9 | 56429 | OSAS | Barrett's esophagus | 1.76E-08 |
| rs11792928 | T | C | 0.0976628 | 0.00261708 | 0.29477 | 0.339048 | 0.016616 | 9 | 56429 | OSAS | Barrett's esophagus | 4.16E-09 |
| rs13195040 | G | A | -0.162354 | -0.0178891 | 0.114653 | 0.0495037 | 0.0245982 | 6 | 56429 | OSAS | Barrett's esophagus | 4.10E-11 |
| rs1868915 | A | C | 0.0892647 | 0.00317789 | 0.586576 | 0.634973 | 0.0153358 | 2 | 56429 | OSAS | Barrett's esophagus | 5.86E-09 |
| rs2861695 | G | A | -0.107195 | -0.0141162 | 0.806143 | 0.807875 | 0.0188427 | 2 | 56429 | OSAS | Barrett's esophagus | 1.28E-08 |
| rs3072 | C | T | 0.110363 | 0.0113053 | 0.367458 | 0.364979 | 0.015734 | 2 | 56429 | OSAS | Barrett's esophagus | 2.31E-12 |
| rs622217 | C | T | -0.0911286 | -0.00365652 | 0.481812 | 0.502134 | 0.0150412 | 6 | 56429 | OSAS | Barrett's esophagus | 1.37E-09 |
| rs739414 | T | C | -0.101127 | -0.00655314 | 0.741878 | 0.72728 | 0.0176328 | 16 | 56429 | OSAS | Barrett's esophagus | 9.74E-09 |
| rs8102046 | G | T | -0.0917291 | 0.00304463 | 0.556026 | 0.687528 | 0.0151203 | 19 | 56429 | OSAS | Barrett's esophagus | 1.31E-09 |

**TableS2** **|**  **Comprehensive details of the SNPs utilized in the MR analysis of BE on OSAS**
